# Immigrants and their children assimilate into US society and the US economy, both in the past and today

**DOI:** 10.1093/pnasnexus/pgae344

**Published:** 2024-10-01

**Authors:** Ran Abramitzky, Leah Boustan

**Affiliations:** Department of Economics, Stanford University, Stanford, CA 94305, USA; National Bureau of Economic Research, Cambridge, MA 02138, USA; Department of Economics, Princeton University, Princeton, NJ 08544, USA; National Bureau of Economic Research, Cambridge, MA 02138, USA

## Abstract

We contribute to the public debate on immigration policy in the United States by providing a long-term, empirical perspective. We develop a novel method of linking individuals across historical Census waves to trace the lives of millions of immigrants in the past and compare their outcomes with immigrants today. We document that upward mobility is just as possible for immigrants today as it was in the early 20th century, and that children of immigrant parents catch up to and frequently exceed the economic outcomes of the children of US-born parents. By our measures, immigrants as a group are no more likely to be incarcerated than those born in the United States, and they assimilate into American culture today at rates comparable to historical standards. Attitudes toward immigrants today are more positive than a century ago, albeit more polarized by political party.

**Significance Statement**

Immigration policy plays a key role in determining population growth and demographic change in the United States. Entry policy also contributes to the outcomes of millions of prospective immigrants hoping to reshape their lives. Rigorous empirical work can provide much-needed clarity to the fundamental questions underlying the contentious public debate over immigration policy and offers a more comprehensive framework for understanding the issue in a historical context. Our long-running work to match individuals across historical US Censuses allows us to trace out trends in immigration since the late 19th century. We find that the children of immigrants continue to catch up to their peers with US-born parents now as in the past, and that the new waves of immigrants incorporate themselves into US economic and cultural life just as well as their historical counterparts. These core findings suggest that policymakers should take the long-run view when considering the immigration system and eschew sensationalized stories in favor of persistent economic and social dynamics.

## Introduction

Contemporary discussions of immigration and immigration policy are often rooted in anecdotes and myths. For example, we often hear a nostalgic view that European immigrants assimilated quickly in the past, while new immigrant groups today do not even attempt to assimilate. The rigorous empirical work conducted by economic historians and other social scientists allows us to subject this conventional wisdom to careful scrutiny.

In our own work, we set out to answer the following questions: (1) Did immigrants benefit from higher rates of upward mobility in the past, in contrast to modern immigrants who struggle to get ahead? What about the children of immigrants? (2) Are today's immigrants less likely to try to integrate into American society than past immigrants? (3) Are attitudes toward immigrants today more negative than in the past? (4) Are today's immigrants more likely to commit crimes than past immigrants?

In our pursuit of answers to these policy-imperative questions, we undertook extensive data collection and linking efforts to track immigrants across time in 2 periods: the late 19th and early 20th century, and 1980 to the present. We developed automated linking methods to match individuals over time across historical US censuses (1, 2). We were able to follow millions of immigrants from their first years in the United States and observe where they came from, where they worked, how much they earned, whether they had children, how their children’s outcomes compared to their own, whether they assimilated into American society, and whether they were more or less likely than the US-born to be incarcerated for a crime. To compare past and present, we combined this historical data with modern tax records provided by Opportunity Insights.

Overall, we find that the “American Dream” for immigrants is just as alive now as it was 100 years ago, yet this dream has never been a quick move from “rags to riches.” The tale of immigrant success has always been a 3-part novel rather than a short story. In the first part, immigrants double their income by leaving home countries that are often poorer than the United States. In the second part, immigrants who arrive with few skills often continue to work in low-paying jobs throughout their lives. And in the third part, the children of immigrants from almost every country move up the economic ladder into the middle class and beyond, catching up to the earnings of the children of US-born parents. This story is just as true today as it was in the past. Furthermore, as a group, immigrants have always been less likely to be incarcerated than those born in the United States. Perhaps because of these positive outcomes, attitudes toward immigrants today are more positive than at any other period in the last 150 years, albeit more polarized than ever by political party.

In our book, *Streets of Gold: America's Untold Story of Immigrant Success* (3), we explore all these issues in depth. In the following, we focus on the most important findings that we uncovered in our pursuit to build a more complete picture of the history of immigration. Our work builds on generations of scholars in economics, sociology, and history who have studied immigration to the United States. We only refer to a few of these studies here. We do not include a comprehensive bibliography in this short piece but refer interested readers to the references included in Abramitzky and Boustan (3).

Relative to this large literature, our contribution lies mainly in using novel data to follow large numbers of immigrant families over time and in comparing immigrants’ outcomes in the past and the present. We are thus able to synthesize our findings into a coherent, comprehensive story of immigration in the United States over the last century. For a detailed statement on immigrant integration in the modern period, see the National Academy of Sciences report (edited by Waters and Pineau) (4).

## Immigration then and now

There is a widely held belief that immigrant entry as a share of the US population has reached unprecedented levels. This is simply not true. Although the share of foreign-born in the US population has more than doubled since mid-century, when immigration policy was most austere, the current share of the population born abroad is comparable to the 1850 to 1910 period. Both then and now, about 1 in 7 residents, or 14% of the population, were foreign-born (5).

Although overall immigration levels remain comparable to the 1850 to 1910 period, significant changes have occurred in the ethnic profile of immigrants. In the past, most immigrants (>90%) hailed from Europe. Now, immigrants from Mexico and several Asian countries constitute a majority. Yet, despite major shifts in countries of origin, as well as in legal regime and economic context, we find that immigrants then and now follow similar paths, both in their outcomes and their efforts and ability to assimilate.

## Immigrants’ economic outcomes

Both in the past and today, immigrants have largely moved to the United States to improve their well-being. By 1900, Norwegians who immigrated to the United States earned twice as much as their brothers who remained behind (6). Over 100 years later, Mexican immigrants at least double their old incomes (7).

Do immigrants fare as well as their US-born peers? Myths suggest that early immigrants caught up to locals with ease, but modern immigrants perform poorly. Once again, this view does not hold up in the data. Comparing immigrants with people born in the United States in the same stages of life suggests that both parts of the “rags to riches in the past” story are wrong. First, both in the past and today, immigrants did not necessarily start in rags. Immigrants from some countries arrived with marketable skills and held high-paying jobs upon arrival on average. Second, when immigrants did arrive in rags, they did not move quickly to riches. Rather, many immigrants who came with fewer skills continued to work in manual labor their entire lives and never made it into white-collar professions (8).^a^

Recent work by Villarreal and Tamborini (26) shows that the children of some immigrant groups today—particularly second-generation Hispanic and Black immigrants—do not converge with the earnings of children of US-born parents in the modern data. Although these findings point to existing racial and ethnic inequalities that certainly exist in the United States, they do not directly address the issue of intergenerational mobility of immigrants—that is, do children of immigrants do systematically better than their parents compared to children of people born in the United States and their parents, conditional on parents’ socioeconomic status?

In our data, we find that, in fact, children of immigrants from almost all countries do better than the average child of people born in the United States. Immigrants’ children, both then and now, have been able to catch up to children of US-born parents (27)^b^ and exhibit significant upward mobility, even if they grew up in poverty. Using the Census, tax records (from Opportunity Insights) (1, 2), and the General Social Surveys, we measured how children of immigrants fared compared to their peers with US-born parents. Children growing up at the bottom of the income distribution are especially mobile. We focus here on children growing up in the 25th percentile of the income distribution as one example of this pattern. In this group, children of immigrants surpass their parents’ economic position at higher rates than children of US-born parents. This finding holds for every period and almost every country of origin.

**Fig. 1.**
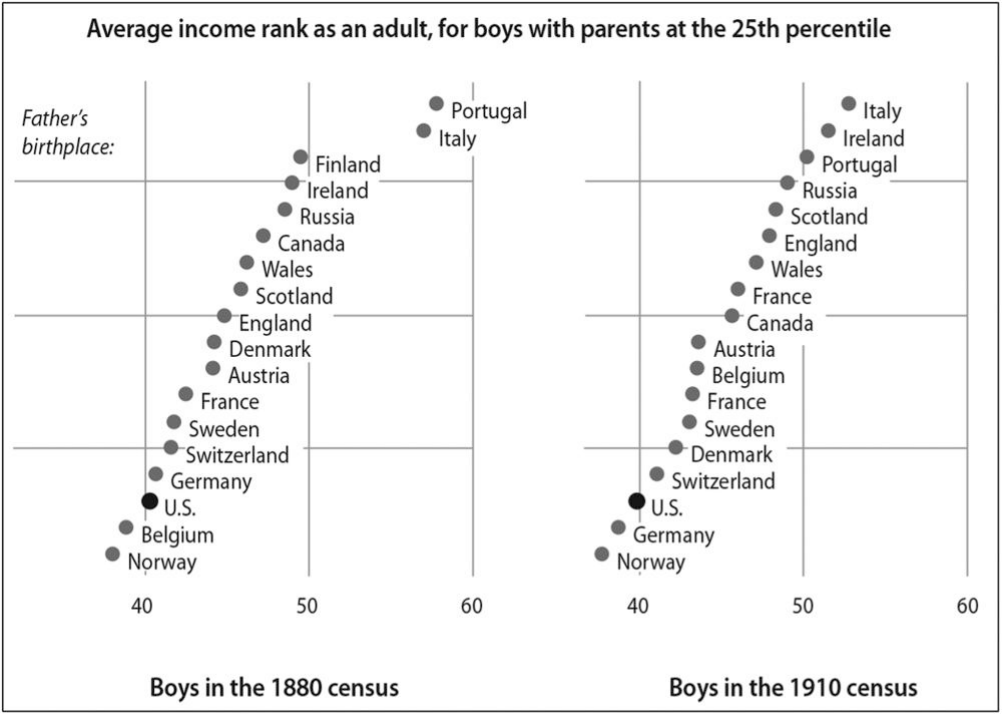
In the past, sons of poor immigrants experienced more economic mobility than the sons of White US-born fathers. These figures compare the income rank of sons raised at the 25th percentile of the income distribution by fathers born in various European countries or in the United States. The sons were observed in their childhood homes in 1880 or 1910, and then in adulthood in 1910 or 1940, where we measure their rank in the income distribution. The underlying data are linked census files. Illustrations by Patti Isaacs, based on Abramitzky et al. (2). From *Streets of Gold* by Leah Boustan and Ran Abramitzky (3) copyright © 2022. Reprinted by permission of PublicAffairs, an imprint of Hachette Book Group, Inc.

In the late 19th and early 20th century, sons born to US-born parents in the 25th percentile of the income distribution rose to the 40th percentile as adults on average (Figure 1). Children of immigrants from all countries other than Belgium and Norway performed significantly better in the 1940 labor market, with children of parents from Portugal and Italy topping the distribution at close to the 60th percentile. A potential limitation of these findings is our reliance on the 1940 Census for children's outcomes, which is at the tail end of the Great Depression. But we document the same pattern for children at home in 1880 and in the labor market in 1910, suggesting that this finding is stable across different economic conditions.

**Fig. 2.**
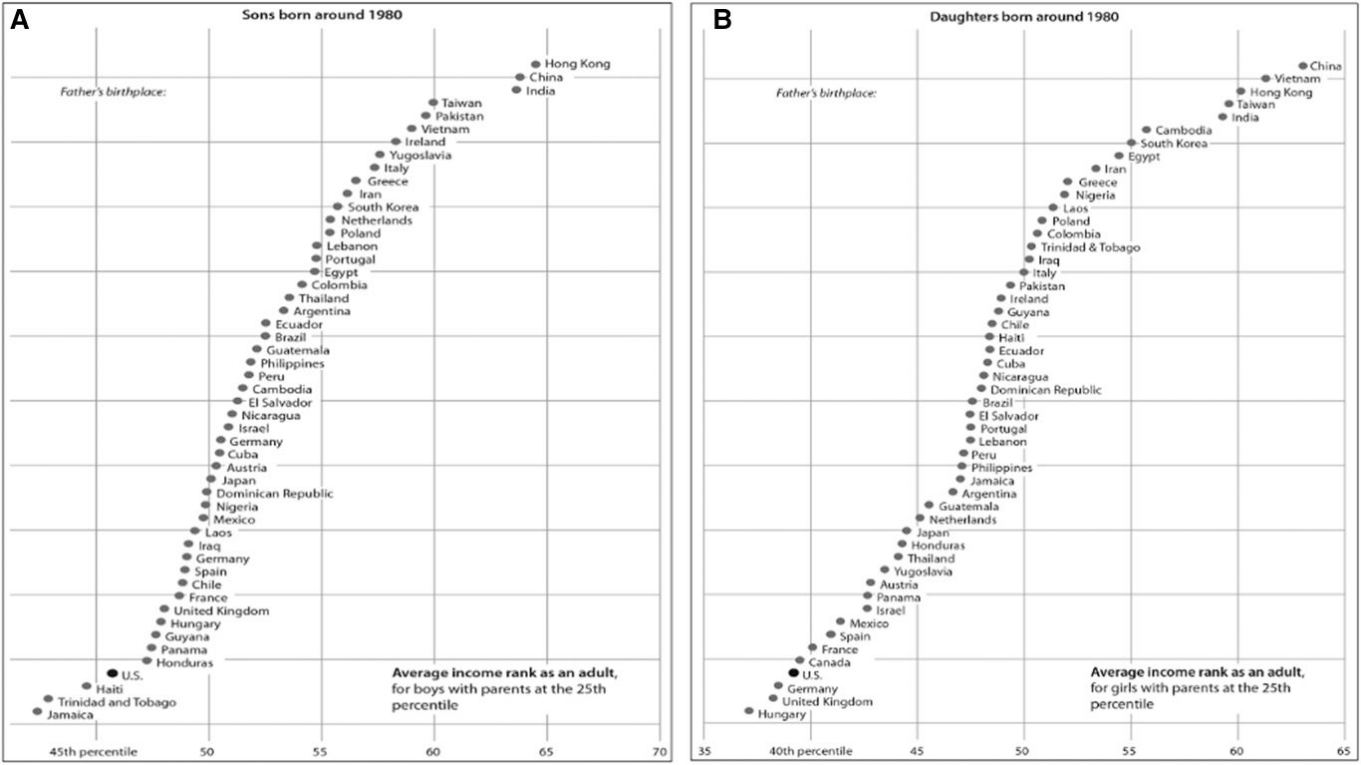
In the modern era, sons (A) and daughters (B) of poor immigrants achieve more economic mobility than the sons and daughters of White US-born fathers. These figures compare the income rank of sons raised at the 25th percentile of the income distribution by fathers born in various countries or in the United States. The sons were born around 1980, and we observe their parents’ outcomes in 1994 to 2000 and the children's outcomes as adults in 2014 to 2015. The data underlying these images come from income tax records provided by the Opportunity Atlas team. Illustrations by Patti Isaacs, based on Abramitzky et al. (31). From *Streets of Gold* by Leah Boustan and Ran Abramitzky (3) copyright © 2022. Reprinted by permission of PublicAffairs, an imprint of Hachette Book Group, Inc.

A hundred years later, this picture is remarkably similar. Sons (daughters) born in 1980 to US-born parents in the 25th percentile of the income distribution reach just above the 45th (40th) percentile by adulthood (Figure 2). Children of immigrant parents fare better overall, with the exceptions of sons of parents from Haiti, Trinidad and Tobago, and Jamaica and daughters of parents from Germany, the United Kingdom, and Hungary. These children, though, are close to the US average (>40th percentile). Strikingly, children of parents from Hong Kong, China, and India reach, on average, almost the 65th percentile as adults.^c^ One limitation of this comparative work is that the tax records used to study modern immigrant families do not contain undocumented immigrant parents. However, the children in this analysis were born in the early 1980s, before the 1986 Immigration Reform and Control Act provided a pathway to citizenship for many undocumented immigrants, and so most of the parents of these cohorts are likely included in the tax data which was collected at mid-childhood (late 1980s and early 1990s).^d^

Why are the children of immigrant parents able to move up the ladder more quickly than the children of US-born parents? One factor is what economists call a “mismatch” of parents’ jobs with their underlying skills and talents. In other words, some immigrant parents cannot realize their income potential because of barriers such as language^e^ and occupational licensing. If income is low in immigrant households for these reasons, immigrant parents are often able to transmit values and human capital to their children that belie their current socioeconomic status.

Another key factor, at least in the past, was immigrants’ locational choices: immigrants move to opportunity. That is, immigrant parents tended to move to areas that offered greater levels of upward mobility. In the past, for example, immigrants rarely settled in the US South, an agricultural region with low upward mobility for all residents relative to the North. When comparing children raised in similar locations, the difference in upward mobility between the children of immigrants and US-born shrinks significantly (we do not yet have access to household-level data to evaluate the role of geography in the modern period.)

Thus, we find that while immigrants themselves may not catch up with their US-born peers, their children not only close the gap but fare even better than the children of people born in the United States. Politicians focusing on the short-term view of immigrants’ outcomes ignore this long-term trend.

## Immigrant cultural assimilation

Another myth is that immigrants culturally integrate more slowly into the US society today than they did in the past, a claim often attributed to the fact that the majority of immigrants today hail from non-European countries. Does this allegation hold up in the data? Measuring “assimilation effort” is difficult, but we can find some telltale signs in various measures of behavior. We focus first on whether parents name their children more “American-sounding” names.

Unlike many other assimilation measures, the names that parents choose for their children reflect an intentional choice made by immigrants that can be directly tied to their perceived cultural identity. Choosing an American-sounding name may reflect a parent learning more about or coming to embrace American culture, and also entails some cost of losing part of their original cultural identity.^f^ Thus, we explored how child naming patterns change as immigrants spend more time in the United States.

We created a name-foreignness index to identify more foreign- versus American-sounding names based on the differential use of various names in immigrant and US-born populations. We find that immigrants tend to choose less foreign-sounding names for their children as they spend more time in the United States, and they do so to a similar degree in both historical and contemporary eras.^g^ This effect is most pronounced for immigrants from less affluent countries, who initially favor foreign names upon arrival yet are among the quickest to transition toward adopting names that sound more native (48).

Another common facet of immigration rhetoric today is the hostility toward refugees, allegedly a group particularly averse to assimilation.^h^ We used rich retrospective interviews with immigrants who transited through Ellis Island to classify historical immigrants’ reasons for moving to the United States. The Ellis Island Foundation conducted approximately 1,200 in-depth interviews with immigrants who arrived in the United States in the early 20th century. In these interviews, we observed whether individuals immigrated for economic reasons, to unite with family, or because they were refugees fleeing distress. We also used linguistic tools to measure their degree of English proficiency (63, 64).

We find that immigrants who reported leaving Europe in response to war, violence, or persecution (“refugee migrants”) attained higher English proficiency than immigrants from the same countries of origin and religious groups who came to join family or find better job opportunities (“economic migrants”). Thus, by this metric, refugees assimilated better than other types of immigrants, a finding that accords with much of the contemporary work on refugees in the United States today.

## Anti-immigration sentiment

**Fig. 3.**
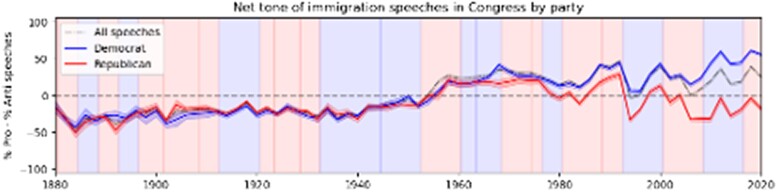
From Card et al. (66).

Anti-immigration sentiment is present in the United States and many other countries that host large shares of the foreign-born. Often, this sentiment is rooted in the belief that immigrants are culturally distant from the natives and are economically “weaker” or less qualified (65). Despite some pockets of anti-immigrant sentiment, attitudes toward immigration today are more positive than ever before in US history, but these views are significantly more polarized by political party (66). We analyzed the language used in congressional speeches over the last 2 centuries: more than 8 million speeches, approximately 200,000 of which pertain to immigration. We then asked human trainers to classify a sample of speeches mentioning immigration as positive, negative, or neutral. We used a large language model to scale this categorization algorithm to the whole corpus of speeches. Prior to approximately 1945, the rhetoric about immigration was largely negative and equally so among Democrats and Republicans (Figure 3). We document a rapid transition to positive attitudes from 1945 to 1965, which persists to this day. At the same time, attitudes have become increasingly polarized by political party, with Republicans becoming increasingly negative and Democrats increasingly positive.

We delved deeper into the analysis of congressional speeches on immigration by categorizing the speeches into 14 different topics. This grouping revealed intriguing shifts in the focus of Democrats and Republicans over time. In the past, politicians were equally likely to address any of these topics, such as crime, labor, and culture, regardless of their political party. Now, Democrats focus more on family and persecution (refugees), while Republicans are significantly more likely to talk about crime and legality. This polarization by topic led us to our next question: is there any evidence that immigrants are more likely to commit crimes than people born in the United States? What do the data say?

## Crime and incarceration rates

Existing correlational research has long shown that immigrants are indeed less likely than people born in the United States to engage in criminal activity, but those studies have been limited in time or geography.^i^ To generalize such findings to the national level and across time, we compiled comprehensive data on incarceration from the Census data from 1870 to 2020, allowing us to compare immigrants with people born in the United States (63, 64).

As a group, immigrants have never been more likely to be incarcerated than people born in the United States, and, in most years, were less likely to be incarcerated. Starting in about 1950, this “immigrant incarceration advantage” started expanding, and by 2020, immigrants were 60% less likely to be incarcerated than people born in the United States. This gap is not driven by the increasing incarceration rates of Black men, as immigrant men are also significantly less likely to be incarcerated compared with White US-born men (30% less). The growing immigrant incarceration gap is also not driven by changes in immigrants’ observable characteristics, namely, their countries of origin, age distribution, race, marital status, state of residence, or educational attainment. We can also rule out that the incarceration gap is mechanically driven by immigrant offenders being more likely to be deported in recent years (and thus not present in incarceration data). The relative decline in immigrant incarceration is entirely driven by an increase in incarceration rates of the less educated people born in the United States. We speculate that less educated immigrants have been relatively insulated from the economic shocks of automation and international trade that have affected the job prospects—and thus perhaps the crime rates—of less educated US-born men.

## Conclusion

Immigration is a highly contested and emotionally salient policy question. Our work brings data and a long-term perspective to the immigration debate. The short-term thinking that politicians tend to apply to immigration policy obscures immigrants’ real successes. Both a century ago and today, immigrants do not move quickly from rags to riches, but their children rise. Our research suggests that it would be a mistake to determine immigration policy based on the belief that immigrants do not integrate. We show that, both now and in the past, immigrants successfully integrate into US society and the US economy. More research is needed on recent undocumented entry at the southern border and the overburdened asylum system. This new immigrant flows at the border might differ from earlier immigrant waves in various ways and might not be immediately captured in government surveys.

## Data Availability

Previously published data (2, 66) were used for this work.

## Notes

a We highlight seminal contributions to this question (9–11) as well as other related papers (12–22). Scholars have documented heterogeneous performance by different national and ethnic groups in the modern period as well: see, for example, Portes and Zhou (23), Portes and Rumbaut (24), and Kasinitz et al. (25).

b Potochnick and Hall (28) documented these patterns by occupation. See Collins and Zimran (29) for an in-depth exploration of intergenerational mobility of the Irish famine migrants. See Kosack and Ward (30) for the analysis of pre–World War II Mexican migrants.

c A potential limitation of our measurement, both for the early and late immigrants, is measuring the outcomes of children at 30 to 50 years of age, which could be considered relatively early in their careers. Inasmuch as income dynamics change after this age, we may be mismeasuring mobility. For instance, Villarreal and Tamborini (26) found that second-generation Hispanic men fall further and further behind White men over their lifetimes. While our measure may not capture such nuance, we rely on Chetty et al. (32), who showed that estimates of mobility in the modern period stabilize when children reach 30 years of age.

d For work on the challenges facing undocumented children or children from families with undocumented members, see Gonzales (33, 34) and Suárez-Orozco et al. (35), among others. A related literature studies the effect of Deferred Action for Childhood Arrivals on immigrant children’s educational and occupational choices (36, 37).

e For evidence on the role of language proficiency on earnings, see Dustmann and Fabbri (38) (United Kingdom), Bleakley and Chin (39) and Heller and Mumma (40) (United States), Arendt et al. (41) (Denmark), and Schmid (42) (Switzerland).

f Another important form of assimilation is interethnic marriages. Alba and Maggio (43) found that ethnoracial assimilation in the United States is led by children of interracial marriages. The most prestigious occupations have become more ethnically and racially diverse through higher shares of mixed-race labor force. Many of them are children of immigrants.

g Fouka (44) documented an increase in Americanized names post–World War I. On the other hand, forcibly restricting German language in Indiana and Ohio after World War I backfired by resulting in more German intramarriages and German naming patterns (45). Saavedra (46) found the same for Japanese Americans after Pearl Harbor. Sue and Telles (47) studied Hispanic immigrant naming practices and found that this group is in a relatively unique position, being able to choose names that are consistent with both their origin-country and US naming practices.

h Research has not yet reached a consensus on how refugees perform compared with economic migrants in the United States. Some find that refugees surpass economic migrants (49, 50), while others find the opposite [e.g., (51, 52)]. Most find that refugees improve their outcomes over time (50–52), see also Evans and Fitzgerald (53). Refugee assimilation is highly dependent on the policies of the receiving countries—for examples from other countries, see Bakker et al. (54) and de Vroome and van Tubergen (55) (the Netherlands), Becker et al. (56) (Germany), Birgier et al. (57) (United States, Sweden, Israel), Donato and Ferris (58) (Canada, Europe, United States), Fasani et al. (59–61) (Europe), and Ruiz and Vargas-Silva (62) (United Kingdom).

i For example, Graif and Sampson (67) showed that, in Chicago, the foreign-born share in a neighborhood is unrelated or negatively related to homicide rates, and areas where residents speak more languages (proxy for immigrant share) are negatively related to homicide rates. In the 1980 and 1990 Census microsamples, immigrants were significantly less likely to be incarcerated than people born in the United States nationwide (68). Using detailed data from Texas, Light et al. (69) found that undocumented immigrants are less likely than people born in the United States to be arrested. Kubrin and Ousey (70) reviewed the existing literature and concluded that a negative relationship exists between immigration and crime.
